# SAS: A Platform of Spike Antigenicity for SARS-CoV-2

**DOI:** 10.3389/fcell.2021.713188

**Published:** 2021-09-20

**Authors:** Lu Zhang, Ruifang Cao, Tiantian Mao, Yuan Wang, Daqing Lv, Liangfu Yang, Yuanyuan Tang, Mengdi Zhou, Yunchao Ling, Guoqing Zhang, Tianyi Qiu, Zhiwei Cao

**Affiliations:** ^1^Department of Gastroenterology, Shanghai 10th People’s Hospital and School of Life Sciences and Technology, Tongji University, Shanghai, China; ^2^CAS Key Laboratory of Computational Biology, Bio-Med Big Data Center, Shanghai Institute of Nutrition and Health, University of Chinese Academy of Sciences, Shanghai, China; ^3^Shanghai Public Health Clinical Center, Fudan University, Shanghai, China

**Keywords:** SARS-CoV-2, spike protein, antigenic resistance, mAb, vaccine

## Abstract

Since the outbreak of SARS-CoV-2, antigenicity concerns continue to linger with emerging mutants. As recent variants have shown decreased reactivity to previously determined monoclonal antibodies (mAbs) or sera, monitoring the antigenicity change of circulating mutants is urgently needed for vaccine effectiveness. Currently, antigenic comparison is mainly carried out by immuno-binding assays. Yet, an online predicting system is highly desirable to complement the targeted experimental tests from the perspective of time and cost. Here, we provided a platform of SAS (Spike protein Antigenicity for SARS-CoV-2), enabling predicting the resistant effect of emerging variants and the dynamic coverage of SARS-CoV-2 antibodies among circulating strains. When being compared to experimental results, SAS prediction obtained the consistency of 100% on 8 mAb-binding tests with detailed epitope covering mutational sites, and 80.3% on 223 anti-serum tests. Moreover, on the latest South Africa escaping strain (B.1.351), SAS predicted a significant resistance to reference strain at multiple mutated epitopes, agreeing well with the vaccine evaluation results. SAS enables auto-updating from GISAID, and the current version collects 867K GISAID strains, 15.4K unique spike (S) variants, and 28 validated and predicted epitope regions that include 339 antigenic sites. Together with the targeted immune-binding experiments, SAS may be helpful to reduce the experimental searching space, indicate the emergence and expansion of antigenic variants, and suggest the dynamic coverage of representative mAbs/vaccines among the latest circulating strains. SAS can be accessed at https://www.biosino.org/sas.

## Introduction

Since the outbreak of the global epidemic of SARS-CoV-2, hundreds of vaccines were designed and several have been successfully developed with S protein as a major antigen (Baden et al., 2021; Dagan et al., 2021; Zhang et al., 2021). Being an ideal vaccine target, the spike glycoprotein could bind with the host receptors such as angiotensin-converting enzyme 2 (ACE2) or mediate direct entry *via* membrane-interacting fusion (Asandei et al., 2020; Hoffmann et al., 2020). So far, the structure complexes between S antigen and corresponding mAbs have been continuously solved at a high resolution, continuing to refresh the understanding of the antigenic positions for this primary antigen. The first binding epitope in S antigen was characterized by a structure complex between the receptor-binding domain (RBD) and a mAb (CR3022) from a convalescent SARS patient (Yuan et al., 2020). Soon, two neutralizing epitope regions adjacent to ACE2 binding sites were identified in the RBD domain *via* non-competing mAbs (B38 and H4) isolated from SARS-CoV-2 patient serum (Wu Y. et al., 2020). Until now, tens of structural epitopes have been derived from S-mAb complexes, while most of them are located in the RBD domain according to the Protein Data Bank (PDB) database (Berman et al., 2000).

Meanwhile, with the mutants continuously arising, several variants have shown antigenic resistance to previously determined mAbs and led to new outbreaks in community. For instance, a new variant B.1.351 in South Africa is refractory to neutralization by multiple mAbs targeting RBD domain, largely owing to the K417N and E484K mutations in the RBD epitopes (Wang et al., 2021). Besides that, recent studies demonstrated that the neutralization ability of mAbs and convalescent or vaccinated sera is decreased against new circulating mutants such as B.1.1.7 and P.1 (Choi et al., 2020; Kemp et al., 2021; McCarthy et al., 2021). As the current vaccines or immune therapeutics were mostly designed based on the initial strain of SARS-CoV-2 from early 2020, it is urgently needed to investigate and monitor the antigenicity shift for the SARS-CoV-2 variants rapidly evolving.

In addition to the classical immuno-binding assays that test the cross-reactivity between targeted S mutants and mAbs one by one, *in silico* suggestion of cross-reactivity is desirable in high throughput and quickly deployable mode. Currently, several tools or servers have been made endeavoring in this direction. Some aim to integrate and visualize genomic information of sequence mutants (Liu et al., 2020; Singer et al., 2020), while others focus on drug-related prediction (Kong et al., 2020; Shi et al., 2020). For antigenicity analysis, two websites, CoV-AbDab (Raybould et al., 2020) and COVIDep (Ahmed et al., 2020), were designed to simply collect either epitopes, or corresponding antibodies, respectively. Summarized from above, none of them enable the cross-reactivity or antigenic resistance prediction between mutated variants, which is critically important to evaluate the effectiveness of mAbs/vaccines previously developed.

Here, a platform of SAS, Spike Antigenicity for SARS-CoV-2, was initiated for this purpose. SAS collects not only validated epitopes from S-mAb complexes in PDB, but also potential antigenic positions predicted by a notable tool of SEPPA 3.0 (Zhou et al., 2019). For each epitope region, antigenic similarity scores were calculated for queried variants against representative S proteins based on the algorithm of CE-BLAST (Qiu et al., 2018). Based on a similarity threshold, the potential antigenic resistance or sensitivity can be auto-suggested for variants recorded in GISAID database (Shu and McCauley, 2017). With future updating, SAS may help to pinpoint those likely escaping strains circulating in community, and indicate the coverage drop of protection for those mAbs or vaccines.

## Results

### Platform Design and Visualization

**FIGURE 1:**
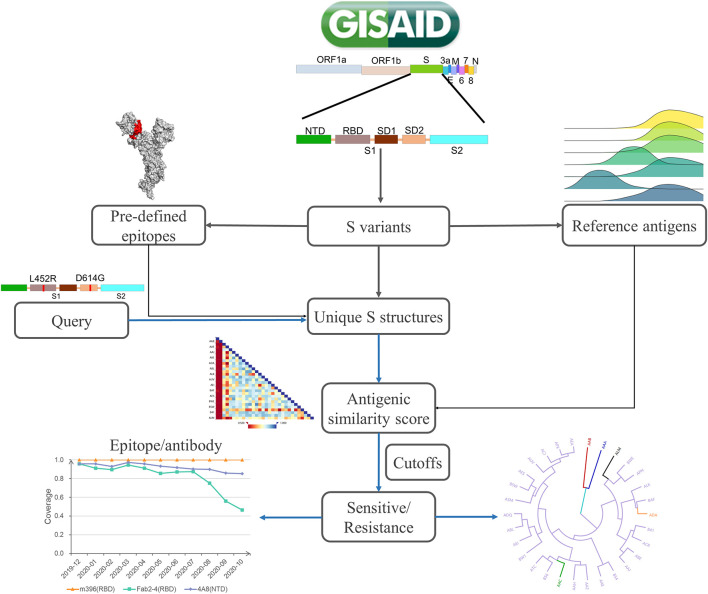
The platform design of SAS. SAS can automatically derive S mutants from the whole-genome sequences collected in GISAID. After quality control, the structure of each S variant is constructed *via* Modeller 9.22. Pre-defined epitope regions are then mapped to modeled structures. For each queried variant, the pairwise antigenic similarity scores are calculated *via* CE-BLAST between query and reference antigens based on individual epitope region. Under a certain cutoff, the antigenic resistance can be suggested and the antigenic clustering tree can be visualized for cross-protection coverage among all reference S antigens.

SAS is designed to automatically collect spike variants from the GISAID database, map amino acid sites to epitope regions, and calculate the antigenic similarity between mutants and reference S proteins on specified epitope regions (Figure 1). For a specific mutant, structures are modeled by Modeller (Webb and Sali, 2016). Then, the collected epitope regions are mapped to variant structures. Furthermore, the antigenic similarity score is calculated by CE-BLAST (Qiu et al., 2018) between the wild and mutated epitope regions. At last, antigenic similarity or escaping are judged according to an adjustable threshold. Be noted that mutational sites out of the epitope regions will not be considered in the calculation. SAS has collected 28 epitope regions, among which 15 were derived from S-Ab complexes in PDB and 13 were predicted by SEPPA 3.0 (Zhou et al., 2019). Altogether, 339 sites have been defined as antigenic among all the 1,273 amino acid positions in S protein (details in Supplementary Table 1). The reference dataset covers monthly representatives and benchmark S proteins. Three spike antigens were set as benchmarks from the coronavirus family, including the SARS-CoV-2 strain (Wu F. et al., 2020), the SARS-CoV strain in NCBI (Marra et al., 2003), and the BatCoV strain (RaTG13) (Zhou et al., 2020). So far, 42 monthly representative mutants have been chosen, covering 71.9% of historical strain isolates with complete S sequence detected. Finally, results of antigenic clustering tree and the heatmap of antigenicity similarity are provided for interactive graphical visualization, together with circulation statistics. Monitoring report is released online when antigenic resistance is detected.

The “Search” function of SAS platform is provided in three ways, including searching “*via mutations*,” “*via GISAID ID*,” and “*via SAS ID*.” For new S variants not yet been covered in SAS, users are allowed to directly submit the .fasta format of spike protein sequence *via* “Predict” section. The input S variant must be a complete sequence with a length above 1,200 AA. Note that, in this case, only the benchmark S of SARS-CoV-2 (SAS ID: AAC) is taken into calculation instead of all the reference spikes, considering the computational cost. However, all pre-defined epitope regions can be scanned.

### High Consistency Between SAS Prediction and Experiment Tests

To test the reliability of SAS predictions, experimental results from two research groups were adopted for comparison including the first and systematic immune-binding experiments of Li’s research (Li et al., 2020) and the comprehensive mAbs/plasma experimental results of J. Greaney’s work (Greaney et al., 2021a,b). Theoretically, the mutants may escape the binding of mAbs if the mutational sites are targeted epitopes of the tested mAbs, or the mutations affect the recognition of tested mAbs. In our paper, the comparison was classified into three levels. Level A refers to the condition when the mutational sites are targeted by tested mAbs. Level B refers to those anti-serum results regarding polyclonal antibodies, and Level C refers to those mAbs results with epitopes unknown or non-overlapping with mutational sites. For Level A, the predictions are made based on the exact epitope regions of the tested mAbs. As for levels B and C, all SAS epitope regions involving the queried mutational sites will be calculated to compare with the experimental results.

In Li’s experiments, 106 S protein mutations were systematically investigated through a panel of 13 neutralizing antibodies and sera from 10 convalescent patients. In total, 35 mutations were clearly listed as antigenic sensitivity or resistance to different tested mAbs or sera. These mutations are distributed over the whole sequence. After checking, 14 mutations fall within the pre-defined epitope regions in SAS, among which 10 were experimentally suggested as already resistant to, or antigenic different from tested mAbs or sera. Tests regarding antibodies B38, CB6, and P2B-2F6 belong to level A. Their epitope regions have been characterized from PDB complexes and collected into SAS as V3, V7, and V9, respectively. Since one mutation may be involved in multiple epitope regions, eight calculations were done, with two predicted to be sensitive and six were resistant. Interestingly, all eight predictions are 100% consistent with experimental conclusions to their corresponding mAbs (Table 1). At level B of sera tests, 14 predictions were done for six mutants, and only one calculation was inconsistent with serum-binding results, resulting in a 92.9% consistency. At level C, three predictions were done for three mutants with a consistency of 50% to experimental results. Prediction is supported by experiments on the Q414E mutant, but failed on N439K, which involved potential de-glycosylation. Experiments on Y508H showed increased sensitivity to mAb B38, but resistance to mAb H104, thus being marked as partial consistency.

**TABLE 1:** Comparison between SAS and experiments on antigenicity measurement.

mAb/sera^a^	Mut	Epi^b^	SAS Id	ASS^c^	SAS^d^	Exp^e^	Cons^f^	Level^g^
B38	A475V	V3	AJY	0.565	V	D	Y	A
CB6		V7		0.584	V	D	Y	
P2B-2F6	L452R	V9	ALM	0.606	V	D	Y	
P2B-2F6	V483A	V9	AA2	0.783	V	D	Y	
B38	F490L	V3	A9P	0.799	V	D	Y	
P2B-2F6		V9		0.735	V	D	Y	
B38	Q409E	V3	AA6	0.826	S	I	Y	
CB6		V7		0.827	S	I	Y	
Sera 10	T478I	V15	ANK	0.628	V	D	Y	B
Sera 10	K458N	V3	AEM	0.674	V	D	Y	
		V4		0.700	V	D	Y	
		V7		0.674	V	D	Y	
Sera 10	V483I	V8	AI6	0.684	V	D	Y	
		V9		0.795	V	D	Y	
		V13		0.738	V	D	Y	
		V15		0.591	V	D	Y	
Sera 10	G446V	V2	AJ2	0.750	V	D	Y	
		V9		0.798	V	D	Y	
		V13		0.664	V	D	Y	
		V15		0.704	V	D	Y	
Sera 3	H519P	V5	ABO	0.919	S	D	N	
Sera 1	N354D	V6	AMA	0.933	S	I	Y	
AB35,157	Q414E	V11	AJX	0.919	S	I	Y	C
B38, H104	Y508H	V1	ACE	0.948	S	I/D	P	
H00S022	N439K	V1	AJI	0.954	S	D	N	

*^*a*^mAb/sera indicates name of tested mAb or sera. Sera 10 indicates sera results from 10 patients; Sera 3 from patient CS2, CS10, and CS86; Sera 1 from patient CS6.*

*^*b*^Number of experimentally validated epitope region in SAS, marked as V1 to V15.*

*^*c*^ASS refers to antigenic similarity score between benchmark S and mutants calculated by CE-BLAST.*

*^*d*^SAS refers to predicted results based on defaulted ASS threshold of 0.8, with V representing varied and S representing similar.*

*^*e*^Exp refers to experimentally validated results through mAb or anti-serum, with D representing decreased antigenicity and I representing increased antigenicity. I/D represents contradicting results between different mAbs.*

*^*f*^Cons refers to consistency between SAS and experiments, with Y representing consistency (Yes), N representing inconsistency (No), and P for partial consistency.*

*^*g*^Levels A, B, and C representing (A) mAbs with known epitope positions; (B) sera containing polyclonal antibodies; and (C) mAbs with unknown epitope positions, respectively.*

In J. Greaney’s work, a mutation scanning method was used to map how the amino acid change in RBD affects antibody binding. Then, this method was applied to human mAbs and polyclonal plasma antibodies from SARS-CoV-2-infected individuals. As no epitope information was available for these mAbs or plasma, their tests correspond to our evaluation of levels B and C. At level B, J. Greaney’s work (Greaney et al., 2021a) displayed the scanning results of 23 mutational sites to plasma of 11 individuals. Among these sites, 17 fell in SAS epitope positions, involving 13 SAS epitope regions. Fifty-five unique S variants containing mutations at the 17 sites were recorded by SAS. To the 11 plasma tests, only 12 mutants were observed with no escaping, while the remaining 43 were reported with at least one escaping out of the 11 plasma and thus being labeled as escaped in J. Greaney’s work. As one site may be covered by different epitope regions, 209 predictions were made on the 17 sites for the 55 variants. As been illustrated in Supplementary Table 2, 166 out of 209 tests (79.4%) were consistent with J. Greaney’s results. At level C of the 10 mAbs, J. Greaney’s work (Greaney et al., 2021b) displayed 36 mutational sites with 29 overlapping with SAS epitope positions. Ninety-nine S variants were recorded in SAS, leading to 327 predictions. Labels of sensitive or escaped were defined similarly to level B according to J. Greaney’s data. As illustrated in Supplementary Table 3, 210 out of 327 tests (64.2%) were supported by J. Greaney’s results.

Overall, SAS gave the consistency of 100% on eight predictions of mAb tests with detailed epitopes covering mutational sites (level A), 80.3% on 223 predictions of anti-serum tests (level B), and 64.2% on 330 predictions of mAbs, but with epitopes unknown or non-overlapping with mutational sites (level C).

### Case Study

SAS allows users to query either mutational sites or a unique variant of spike sequence (SAS ID). In our dataset, one mutational site may be involved by multiple epitope regions, while one validated epitope region corresponds to at least one mAb. Thus, both mutations and epitope regions should be specified in order to obtain the potential sensitivity/resistance of a mAb to the queried variant.

**FIGURE 2:**
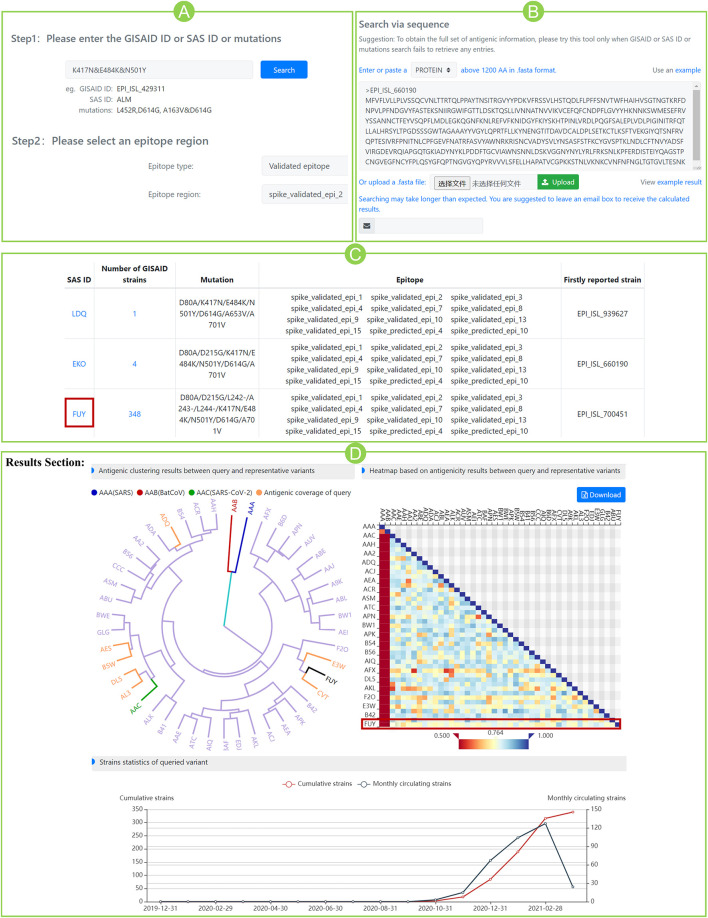
Case study for community circulating strain B.1.351 from South Africa. **(A)** Submit mutations K417N&E484K&N501Y and specify a pre-defined epitope region. **(B)** Alternatively, submit the spike sequence in “predict” section. **(C)** Variants that contain the queried mutations. For each variant, the number of GISAID strain, mutation information, epitope regions involving queried mutations, and the firstly reported strain were listed in the table. **(D)** Results section of variant “FUY”. The antigenicity clustering tree (left) shows the potential sensitivity (in yellow) or resistance (in purple) of queried variant to all representative variants. The three benchmark variants are marked red (AAA), blue (AAB), and green (AAC), respectively. The heatmap (right) shows the antigenic scores between query and representative variants (marked in red box). The line chart (bottom) indicates the dynamic circulation statistics of queried variant. The blue line shows the number of monthly detected circulating strains and the red line shows the cumulative strains.

For the case study, the community circulating strain B.1.351 from South Africa, with RBD mutations of K417N, E484K, and N501Y, was taken as an example to illustrate the SAS application (Figure 2). Step 1: Query input: mutation “K417N&E484K&N501Y” is input as a query (Figure 2A). Alternatively, in “*predict*” section, users can submit the spike sequence with K417N&E484K&N501Y mutations (Figure 2B). Step 2: Epitope selection: for the convenience of user’s selection, all variants of SAS IDs are displayed in a table together with available epitope regions involving the queried mutational sites. Based on the above, users can select interested epitope regions, and click hyperlinks of interested S variants to continue. It can be seen that the above queried mutations are involved in nine validated epitope regions of 2, 3, 4, 7, 8, 9, 10, 13, and 15 (Figure 2C). Here, we selected the validated epitope region 2 and clicked SAS ID of “FUY” as example.

The section “Results” displays antigenicity clustering tree, heatmap of antigenic scores, and dynamic circulation statistics of the queried variant (Figure 2D). The clustering tree indicated the overall potential sensitivity (in yellow) or resistance (in purple) of queried variant to all reference variants. The three benchmarks are marked as well. In the heatmap of Figure 2D, variant FUY seems to have gained reduced antigenicity (score < 0.8) against not only the benchmark AAC variant (ASS score 0.764), but also the majority of reference variants. Furthermore, circulation statistics showed that it was firstly reported in October 2020 in South Africa (hCoV-19/South Africa/NHLS-UCT-GS-0644/2020) and became circulated after then.

More antigenicity scores of additional eight epitope regions can be found in Supplementary Table 4. Interestingly, on all nine epitopes, the antigenic scores of FUY against AAC are all below 0.8, indicating the significantly reduced antigenicity. Those scores even go below 0.7 at epitopes 4, 10, 13, and 15, indicating the varied antigenicity. Our prediction aligned well with the latest reports that mRNA vaccines showed reduced effectiveness on circulating strains of B.1.351 in South Africa (Wang et al., 2021), and this was mainly caused by the mutations of K417N&E484K&N501Y (Garcia-Beltran et al., 2021). Meanwhile, the scores of other epitope regions not involving queried mutations, such as epitopes 5, 6, 11, and 12, all give high similarity scores above 0.9. The above agreement indicates the potential usefulness of SAS in suggesting cross-reactivity for SARS-CoV-2.

### Monitoring Reports

**FIGURE 3:**
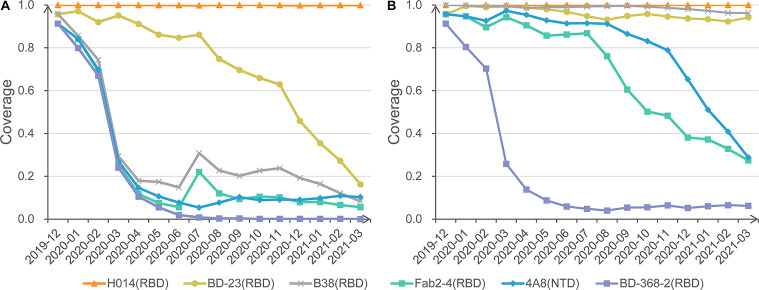
Protection coverage of exemplary anti-S mAbs at different cutoffs. **(A)** Monthly protection coverage of six mAbs with the cutoff of 0.8. **(B)** Monthly protection coverage of six mAbs with the cutoff of 0.7.

The monitoring section gives two reports. One is about the most resistant variant accumulated so far to the AAC variant for each epitope region or corresponding mAb. Another is the dynamic protection coverage of each mAb among the monthly circulating variants in Figure 3.

Also, we invite users to change different cutoffs of antigenic resistance and visualize the dynamic coverage for each mAb. For example, under the strict cutoff of 0.8, mAbs H014 shows complete activity to all circulating strains. In contrast, BD23 shows a declining trend of coverage from 95.7% in December 2019 to 16.2% in March 2021. Other mAbs, such as Fab2-4 and B38, exhibit significantly rugged but decreasing coverage with time moving (Figure 3A). Further investigation found that the curve bump in July is associated with the transient emergence of S477N/D614G cluster. Additional mAbs, CR3022, S309, m396, and EY6A, present overlapping curves with H014 (not displayed).

When a looser cutoff of 0.7 is switched, the theoretical coverage of three mAbs (B38, Fab2-4, and 4A8) increase sharply, though a falling trend remains for Fab2-4 and 4A8 (Figure 3B). No matter how, BD-368-2 remains the lowest coverage based on SAS prediction. Careful checking found that BD-368-2 targets a long and flexible loop region in the RBD domain (Supplementary Figure 1). Indeed, in a neutralization test of four variants, three exhibited resistance to BD-368-2, and only one (25%) was detected as sensitive (Du et al., 2020).

## Discussion

With the increasing spread of SARS-CoV-2, evaluating the cross-neutralization among emerging variants becomes critically important to vaccine development. While experimental screening is classical but time-consuming, we constructed an online prediction platform as a complement to reduce experimental space, by suggesting antigenic resistance of emerging mutants and the potential antigenic coverage of representative mAbs. SAS can automatically update and detect the potential antigenic variants, and give alert if such variants spread in community over time.

SAS stands as the first platform focusing on cross-neutralization auto-prediction for SARS-CoV-2, which is enabled based on structural epitope mapping and further epitope comparison to infer antigenic similarity/resistance. In this work, pre-defined epitope regions are primary information to calculate the antigenic similarity. Currently, the validated antigenic regions are determined from complexes in PDB (Berman et al., 2000), and most of them are located in the RBD domain. Additional epitopes were appended through SEPPA 3.0 prediction (Zhou et al., 2019). Several tools are available online for B-cell epitope prediction, such as DiscoTope 2.0 (Kringelum et al., 2012), BepiPred 2.0 (Jespersen et al., 2017), and SEPPA 3.0 (Zhou et al., 2019). The reason to choose SEPPA 3.0 is that, it is the only one designed for glycoprotein antigens and maintains competent performance among peers.

In terms of antigenicity comparison, we chose CE-BLAST method (Qiu et al., 2018). Typical *in silico* peers usually need training from massive accumulation of the historical data, such as the model for influenza virus (Liao et al., 2009; Du et al., 2012), and foot-and-mouth disease virus (FMDV) (Reeve et al., 2010). Whereas for SARS-CoV-2 with limited data of immune-binding assays, only CE-BLAST enables antigenic pre-estimation for newly emerging pathogens. Moreover, the early cross-reactivity predictions from CE-BLAST (Qiu et al., 2020) have been partially verified by later crystallization of spike–mAb complexes between SARS-CoV-2 and SARS-CoV (Cao et al., 2020). In this study, the antigenicity score from CE-BLAST describes the antigenic similarity/resistance between mutated epitopes. It was designed by incorporating both the structural similarity and physicochemical similarity in the micro-environment of a 10-Å shell around the mutational sites to tolerate certain fluctuation (Qiu et al., 2018). Though not being directly correlated with neutralization activity, the antigenicity score here was used as an indicator of relative antigenic relationships between epitopes. In Table 1, the mutation of A475V was suggested with the lowest score by SAS. Interestingly, Li’s comprehensive investigation confirmed that this mutation gained decreased sensitivity to both human sera and multiple neutralizing mAbs (Li et al., 2020). It displayed reduced reactivity to six out of the 13 tested mAbs and demonstrated significant resistance to four well-characterized antibodies (Li et al., 2020). Similarly, L452R gained the second-lowest and was confirmed to be significantly resistant to two antibodies (Li et al., 2020).

The high consistency between SAS prediction and three sets of experimental datasets indicates the promising application of SAS to suggest antigenic variation, particularly for those mAb tests with detailed epitope positions and those anti-serum tests of polyclonal antibodies. The decreased consistency from Level A to Level C is reasonable. At level A, SAS gives a highly reliable prediction as the queried mutational sites are targeted by tested mAbs. At Level B, SAS makes calculation based on multiple epitope regions involving the mutational sites. As anti-serum contains polyclonal antibodies and often targets multiple epitope regions, SAS predictions agree well with anti-serum results. In the case of level C, the lower consistency is expected since the mutational sites may not be recognized by tested mAbs.

In summary, we here constructed a SAS platform focusing on the antigenic resistance prediction for SARS-CoV-2 mutants. In addition to the auto-collecting of spike variants from GISAID, SAS will keep updating the latest epitope regions, additional mAbs, and other antigens and prediction algorithms in the future. Despite that, we would like to remind the limitations when interpreting SAS results: (1) Quaternary organization: spike structures appear as both monomer and trimer states with different epitopes, while current SAS only considers monomer condition. (2) Dynamic conformation: spike owns “up” and “down” conformation, while only the “down” template was considered in the current version. (3) Structure modeling: SAS calculation relies on structure modeling. Though SAS scans 10 Å around each mutational site to accommodate structural fluctuation, prediction accuracy may decrease for those mutant sites far from epitope regions. With future improvement, SAS might help to reduce the experimental screening space for immune-binding tests, recommend the antigenic variant that emerged in community, and suggest the potential coverage of mAbs/vaccines, so as to facilitate vaccine and immune therapeutics design for SARS-CoV-2.

## Materials and Methods

### Data Collection and Pre-defined Epitope Preparation

The whole-genome sequences of circulating SARS-CoV-2 isolates were collected from the GISAID database (Shu and McCauley, 2017) (GISAID accession numbers recorded in Supplementary Table 5). By aligning to the reference sequence of SARS-CoV-2 (Wu F. et al., 2020), compete spike protein-coding regions were extracted for each isolate and further translated to amino acid sequences. The benchmarking S proteins were collected from the NCBI protein database (Sayers et al., 2020), which include SARS-CoV (Marra et al., 2003), SARS-CoV-2 (Wu F. et al., 2020), and the potential bat original strain of BatCoV (RaTG13) (Zhou et al., 2020).

Validated epitope regions were derived from the S protein–antibody complexes of both SARS-CoV and SARS-CoV-2 released in PDB (Berman et al., 2000). For each complex structure, those residues on the S protein with the nearest atom distance toward the corresponding antibodies less than 4.0 Å were defined as the epitope residues. Then, small epitopes with less than 10 residues were removed. Furthermore, similar epitopes that bind with the same mAb were merged as one epitope region. Finally, 15 validated epitopes were determined.

Predicted epitope regions were obtained through the prediction of SEPPA 3.0 (Zhou et al., 2019) based on the structure of SARS-CoV-2 spike glycoprotein (PDB ID: 6VXX, Chain: A) (Walls et al., 2020). By setting the immune host as “*Homo sapiens*” and subcellular localization as “*Membrane*”, 358 residues were predicted as potential epitope residues. Among them, successive residues within the 10 Å range were defined as a continuous epitope region. After removing small epitope with less than 10 residues, 13 predicted epitope regions remained. Detailed information of both validated epitopes and predicted epitopes can be found in Supplementary Table 1.

### Data Processing Pipeline of SAS

SAS was designed to automatically obtain the SARS-CoV-2 spike proteins and provide the latest antigenic similarity results of the current available unique S variants (Figure 1). Initially, the nucleic acid sequences of the S protein were derived from the whole-genome sequence (bases 21,563–25,384) released in GISAID and then translated into amino acid sequences. After quality control including removing (1) sequences from species other than *Homo sapiens*, (2) sequences with ambiguous amino acids such as “*X*,” and (3) redundant sequences, the dataset for unique S variants can be constructed. Then, by setting the structure of SARS-CoV-2 spike protein (PDB ID: 6VXX, Chain: A) as a template, protein structure modeling was performed for each S variant *via* Modeller 9.22 (Webb and Sali, 2016) to obtain the homology structure, which represents the basic unit in SAS with identical SAS ID.

Currently, SAS ID is encoded in three characters, with each character coded by a capital letter from A to Z or a number from 1 to 9. Besides, three unique S variants with the largest abundance representing the dominant circulation in community will be automatically derived monthly as representative S variants. Specifically, the S protein of all circulating SARS-CoV-2 strains within 1 month will be mapped to the unique S variants. The top three S variants with the largest population among circulating strains will be selected as representative S variants. Note that if the top abundant S variant has already been included in the representatives for previous months, sub-abundant ones will be considered, until three new representative S variants were obtained.

For each unique S variant, 28 pre-defined epitope regions involving 15 experimentally validated epitopes named *spike_validated_epi_1* to *spike_validated_epi_15* (Hwang et al., 2006; Prabakaran et al., 2006; Pak et al., 2009; Pinto et al., 2020; Wrapp et al., 2020; Wu Y. et al., 2020; Yuan et al., 2020) and 13 computationally predicted epitopes named *spike_predicted_epi_1* to *spike_predicted_epi_13* were mapped to the corresponding S protein structures *via* sequence alignment tool of BLAST (Camacho et al., 2009). Furthermore, the pairwise antigenic similarity score between each unique S variant and reference antigens on the pre-defined epitope region was calculated through CE-BLAST (Qiu et al., 2018) (see section “Calculation of SAS Score”). Moreover, the antigenic clustering was obtained from the hierarchical clustering based on the scoring matrix of antigenic similarity. Finally, the antigenic clustering tree and the antigenicity heatmap could be obtained to provide visualized antigenic similarity among query S variant, monthly representative S variants, and the benchmarking S antigens in each pre-defined epitope.

Moreover, SAS will automatically calculate the antigenic similarity of new S variants against the S antigens of benchmarking SARS-CoV-2 on all pre-defined epitopes. The lowest antigenic similarity of S variant on each pre-defined epitope will be selected to form the monitoring report after each updating of S variants. The rationale of the scheme is set as, the higher the score, the less possible for the mutants to escape. Thus, the alert is given at four levels, including the following: (1) Blue: antigenic score ≥ 0.8, similar antigenicity; (2) Yellow: 0.7 ≤ antigenic score < 0.8, reduced antigenicity; (3) Orange: 0.6 ≤ antigenic score < 0.7, varied antigenicity; (4) Red: antigenic score < 0.6, drifted antigenicity. The current thresholds of antigenic similarity are empirical cutoff derived from 679 PDB immune complexes. Basically, the two epitopes are defined as antigenic similar (≥0.8) when their corresponding antibodies share over 98% CDR sequence similarity based on CE-BLAST (Qiu et al., 2018). While under a similarity score of 0.7, epitopes in the same antigen can be roughly detected.

Note that for a new sequence of S protein submitted to SAS, the quality of the query will be checked first. Exclusion criteria include (1) sequence length less than 1,200 amino acids from N-terminal, (2) identities to benchmarking SARS-CoV-2 S protein less than 90%, and (3) the sequence contains ambiguous amino acids other than 20 normal amino acids. After quality checking, SAS will automatically construct the S protein structure based on the template of the SARS-CoV-2 spike protein (PDB ID: 6VXX, chain A) *via* Modeller 9.22 (Webb and Sali, 2016). Then, the antigenic similarity score between query and the benchmarking S antigens of SARS-CoV-2 in all pre-defined epitope regions will be calculated through the algorithm of CE-BLAST (Qiu et al., 2018; Supplementary Figure 2).

### Calculation of SAS Score

Similarity score in SAS was calculated based on the algorithm of CE-BLAST (Qiu et al., 2018), which took full consideration of the amino acid layout and physicochemical micro-environment in the structural epitopes. CE-BLAST model was designed into three steps: (1) deriving a group of fingerprints for each structural epitope, (2) aligning the conformational epitopes according to their fingerprints, and (3) scoring the similarity according to the epitope alignment. More technical details can be found in CE-BLAST (Qiu et al., 2018).

### Platform Architecture

The platform of SAS is constructed based on the Spring boot^1^, a mature and convention-over-configuration Model-View-Controller (MVC) framework that embedded Tomcat service^2^ on a Centos Linux server (version 7.7). The Metadata of SAS is stored in MongoDB^3^, a general-purpose, document-based, distributed database. The front-end interfaces are built using JavaScript^4^ to provide responsive and user-friendly web pages. Also, RabbitMQ is used for real-time communication between modules and modules that were packaged into docker to ensure flexibility in website deployment. The pipeline of the tool is based on Luigi, a Python package that could be used to build complex pipelines of batch jobs. Phylotree^5^ was used for phylogenetic tree drawing and echarts^6^ was used for MDS plot drawing. SAS uses a distributed, in-memory search engine, Elasticsearch engine^7^, to realize fast and flexible text search.

## Data Availability Statement

The datasets presented in this study can be found in online repositories. The names of the repository/repositories and accession number(s) can be found in the article/Supplementary Material.

## Author Contributions

LZ, RC, TM, YW, and MZ collected the data. RC, DL, LY, YT, YL, and GZ constructed the web server. LZ, TQ, and ZC designed the experiments and wrote the manuscript. GZ, TQ, and ZC co-supervised the whole project. All authors contributed to the article and approved the submitted version.

## Conflict of Interest

The authors declare that the research was conducted in the absence of any commercial or financial relationships that could be construed as a potential conflict of interest.

## Publisher’s Note

All claims expressed in this article are solely those of the authors and do not necessarily represent those of their affiliated organizations, or those of the publisher, the editors and the reviewers. Any product that may be evaluated in this article, or claim that may be made by its manufacturer, is not guaranteed or endorsed by the publisher.

## Footnotes

1
http://spring.io

2
https://tomcat.apache.org

3
http://www.mongodb.com

4
https://www.javascript.com

5
http://phylotree.hyphy.org

6
https://echarts.apache.org

7
https://www.elastic.co/products/elasticsearch
