# Supplementary material for: SAS: A Platform of Spike Antigenicity for SARS-CoV-2
Source: Front Cell Dev Biol. 2021 Sep 20;9:713188. doi: 10.3389/fcell.2021.713188 (PMC8488377; doi:10.3389/fcell.2021.713188)
Supplement: Supplementary file 2 [file Data_Sheet_1.docx]

**Supplementary Materials**

**Supplementary Figures**


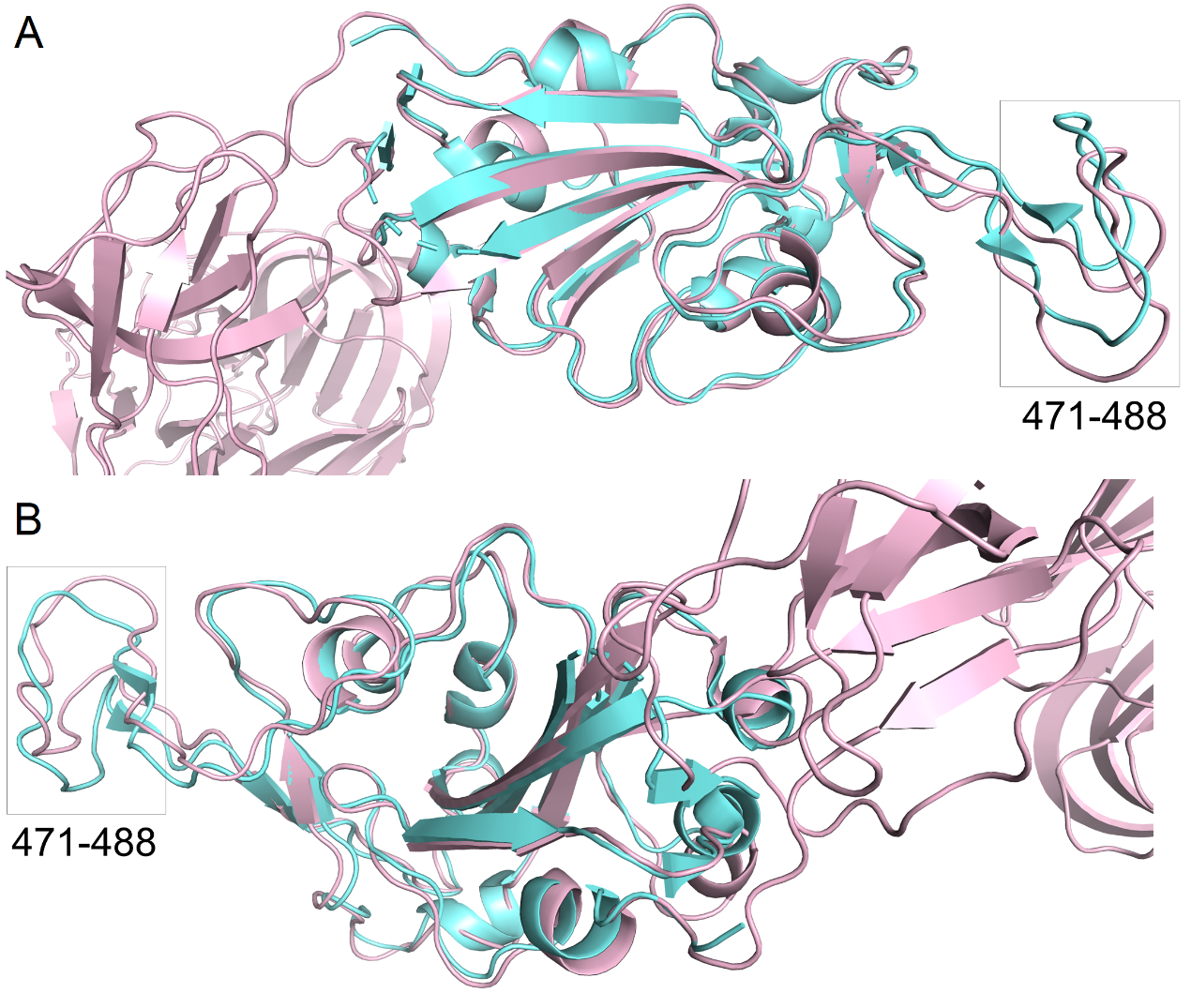


**Supplementary Figure 1.** Structure alignment between two crystalized spike protein structures. (A) Structure alignment between the spike protein (PDB ID: 6ZGG, Chain: A) and the RBD region of spike protein (PDB ID: 7CHB, Chain: R). The flexible loop region from site 471 to site 488 was marked in boxes. (B) Structure alignment rotating 180 degrees of Figure S1A.


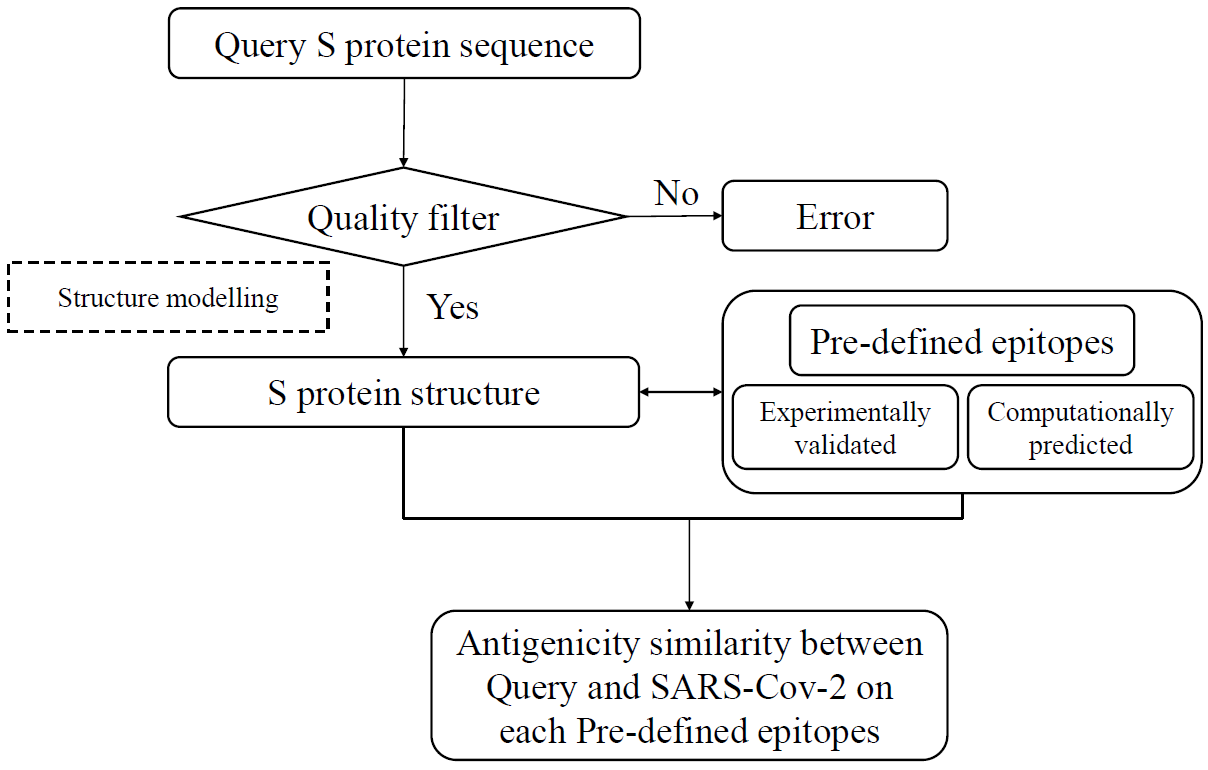


**Supplementary Figure 2**. The pipeline of SAS for prediction function. For any uploaded S protein sequence, SAS will go through a quality filter. For any sequence pass the quality check, S protein structure will be constructed. The antigenicity similarity score between query and the S protein of benchmarking SARS-CoV-2 will be calculated through all pre-defined epitopes.

**Supplementary Tables**

**Supplementary Table 1. The detailed information of 28 pre-defined epitopes.**

| Pre-defined epitopes | Epitope position | PDB ID^a^ |
| --- | --- | --- |
| spike_validated_epi_1 | 372,375,376,378,403,404,405,408,437,439,440,449,496,498,499,500,501,502,503,504,505,506,508 | 2DD8,3BGF |
| spike_validated_epi_2 | 446,449,484,485,486,489,490,492,493,494,496,498,501,505 | 7BYR |
| spike_validated_epi_3 | 403,405,409,415,416,417,420,421,453,455,456,457,458,460,473,475,476,477,486,487,489,490,493,495,496,498,500,501,502,505 | 7BZ5 |
| spike_validated_epi_4 | 403,405,408,415,416,417,421,453,455,456,457,458,459,460,473,474,475,476,477,486,487,489,498,500,501,502,504,505 | 6XCM |
| spike_validated_epi_5 | 369,370,371,372,374,376,377,378,379,380,381,382,383,384,385,386,390,392,428,429,430,515,516,517,518,519 | 6W41,6YLA,6YM0 |
| spike_validated_epi_6 | 334,335,337,339,340,343,344,345,346,354,356,357,359,360,361,441,509 | 6WPS,6WPT |
| spike_validated_epi_7 | 403,405,406,408,409,415,416,417,420,421,455,456,457,458,460,473,474,475,476,477,486,487,489,493,495,502,504,505 | 7C01 |
| spike_validated_epi_8 | 449,453,455,456,483,484,485,486,487,489,490,492,493,494 | 6XEY |
| spike_validated_epi_9 | 346,444,446,447,448,449,450,452,483,484,485,490,494 | 7BWJ |
| spike_validated_epi_10 | 417,453,455,456,484,485,486,487,488,489,493 | 6XDG |
| spike_validated_epi_11 | 369,374,375,376,377,378,379,380,381,382,383,384,385,386,388,389,390,392,411,412,413,414,427,428,429,430,517 | 6ZER,6ZCZ,6ZFO,6ZDH,6ZDG |
| spike_validated_epi_12 | 369,372,373,374,375,376,377,378,379,380,385,405,407,408,411,412,503,504 | 7CAH |
| spike_validated_epi_13 | 446,449,456,475,483,484,485,486,487,489,493 | 7JMP |
| spike_validated_epi_14 | 144,145,146,147,150,152,245,246,247,248,249 | 7C2L |
| spike_validated_epi_15 | 444,446,447,449,450,452,470,478,479,481,482,483,484,485,490 | 7CHH |
| spike_predicted_epi_1 | 30,32,33,34,56,57,58,59,60,61,63,191,217,218,219,266,267,293 | Predicted |
| spike_predicted_epi_2 | 38,39,40,41,42,43,44,45,49,51,52,53,204,206,220,221,222,224,225,278,279,280,282,283,284,285,286,287,288 | Predicted |
| spike_predicted_epi_3 | 50,273,274,291,298,301,302,303,304,305 | Predicted |
| spike_predicted_epi_4 | 65,80,81,82,83,84,85,86,104,108,110,199,231,233,234,235,236,237,238,239,269 | Predicted |
| spike_predicted_epi_5 | 109,111,112,113,114,115,116,132,133,134,135,136,137,139 | Predicted |
| spike_predicted_epi_6 | 327,328,329,388,389,528,529,530,531,532,533,534,542,544,545,546,547,578,579,580 | Predicted |
| spike_predicted_epi_7 | 341,342,344,345,346,347,348,349,350,351,399,401,402,436,442,448,450,451,454,509 | Predicted |
| spike_predicted_epi_8 | 600,601,603,604,605,663,673,674,675,676,689,690,691,692 | Predicted |
| spike_predicted_epi_9 | 654,655,656,657,658,660,671,693,695,696 | Predicted |
| spike_predicted_epi_10 | 659,661,662,669,697,698,699,700,701,702 | Predicted |
| spike_predicted_epi_11 | 723,724,725,726,938,1028,1041,1042,1043,1044,1045,1047,1048,1068 | Predicted |
| spike_predicted_epi_12 | 727,728,729,730,731,777,778,782,944,945,946,947,948,949,951,1017,1018,1019,1020,1021,1022,1023,1024,1026,1027,1056,1057,1058,1059 | Predicted |
| spike_predicted_epi_13 | 884,885,886,887,888,889,890,891,892,893,894,895,1035 | Predicted |

^a^Validated epitopes are derived from the Protein Data Bank, the corresponding PDB IDs were provided. Predicted epitopes are calculated by SEPPA 3.0.

**Supplementary Table 2. Comparison between SAS and experimental results on plasma.**

^a^ Plasma indicates name of plasma samples that show reduced binding. ^b^ Number of experimentally validated epitope region in SAS, marked as V1 to V15. ^c^ ASS refers to antigenic similarity score between benchmark S and mutants calculated by CE-BLAST. ^d^ SAS refers to predicted results based on defaulted ASS threshold of 0.8, with V representing varied and S similar. ^e^ Exp refers to experimentally validated escape for plasma, with E representing escape and nE representing not escape. ^f^ Cons refers to consistence between SAS and experiments, with Y representing consistency (Yes) while N representing inconsistency (No).

**Supplementary Table 3. Comparison between SAS and experimental results on mAbs.**

^a^ mAb indicates name of escaping mAb medicated by the following mutantion. ^b^ Number of experimentally validated epitope region in SAS, marked as V1 to V15. ^c^ ASS refers to antigenic similarity score between benchmark S and mutants calculated by CE-BLAST. ^d^ SAS refers to predicted results based on defaulted ASS threshold of 0.8, with V representing varied and S similar. ^e^ Exp refers to experimentally validated escape for mAb, with E representing escape and nE representing not escape. ^f^ Cons refers to consistence between SAS and experiments, with Y representing consistency (Yes) while N representing inconsistency (No).

**Supplementary Table 4. Antigenicity scores on RBD epitopes between the South Africa variant (SAS ID: FUY) and reference strain (SAS ID: AAC).**

| **Pre-defined epitopes** | **Involved Mutations** | **Antigenic Score** |
| --- | --- | --- |
| spike_validated_epi_2 | 484, 501 | 0.764 |
| spike_validated_epi_3 | 417, 501 | 0.769 |
| spike_validated_epi_4 | 417, 501 | 0.683 |
| spike_validated_epi_5 | N/A | 0.988 |
| spike_validated_epi_6 | N/A | 0.987 |
| spike_validated_epi_7 | 417 | 0.723 |
| spike_validated_epi_8 | 484 | 0.766 |
| spike_validated_epi_9 | 484 | 0.781 |
| spike_validated_epi_10 | 417,484 | 0.518 |
| spike_validated_epi_11 | N/A | 0.986 |
| spike_validated_epi_12 | N/A | 0.986 |
| spike_validated_epi_13 | 484 | 0.647 |
| spike_validated_epi_15 | 484 | 0.612 |

**Supplementary Table 5. The list of GISAID accession numbers.**
